# A nomogram-based radiomics for predicting survival to concurrent chemoradiotherapy in inoperable pancreatic cancer: a dual-center cohort study

**DOI:** 10.3389/fimmu.2025.1655803

**Published:** 2025-10-23

**Authors:** Xin Liu, Ke Su, Shanshan Du, Yanze Li, Peiping Sun, Shucheng Shen, Benzhe Liang, Jian Chen, Rui Liu, Rui Zhang, Heran Wang, Huadong Wang, Yong Yin, Zhenjiang Li

**Affiliations:** ^1^ Department of Radiation Physics, Shandong Cancer Hospital and Institute, Shandong First Medical University and Shandong Academy of Medical Sciences, Jinan, China; ^2^ Department of Gynecological Radiotherapy, Harbin Medical University Cancer Hospital, Harbin, China; ^3^ Department of Radiation Oncology, National Cancer Center/National Clinical Research Center for Cancer/Cancer Hospital, Chinese Academy of Medical Sciences and Peking Union Medical College, Beijing, China; ^4^ Department of Oncology, The Affiliated Hospital of Southwest Medical University, Luzhou, China; ^5^ School of Physics and Electronic Science, Shandong Normal University, Jinan, China; ^6^ Department of Graduate, Shandong First Medical University, Shandong Academy of Medical Sciences, Jinan, China; ^7^ School of Nuclear Science and Technology, University of South China, Hengyang, China; ^8^ Department of Emergency Medicine, Nanjing Drum Tower Hospital, Nanjing Drum Tower Hospital Clinical College of Nanjing University Of Chinese Medicine, Nanjing, China; ^9^ Department of Orthopedics, Shengjing Hospital of China Medical University, Shenyang, Liaoning, China

**Keywords:** pancreatic cancer, machine learning, prognosis, radiology, survival

## Abstract

**Objective:**

This study was designed to explore the value of machine learning-based radiology in predicting overall survival (OS) among patients with inoperable pancreatic cancer (PC) who are undergoing concurrent chemoradiotherapy (CCRT).

**Methods:**

This multicenter study enrolled 342 patients with inoperable PC. Firstly, radiomic features were pre-screened by univariate Cox regression and subsequently used to develop 101 machine-learning–based imaging models. An optimized selection algorithm was applied to these models to derive each patient’s radiomic signature (Rad-score). Secondly, key clinical predictors of OS were identified via LASSO–Cox regression and incorporated into clinical nomogram. Finally, the Rad-score was combined with the independent clinical risk factors to construct clinical–radiomics nomogram.

**Results:**

LASSO–Cox regression identified age, clinical stage, tumor size, and albumin level as independent prognostic factors for OS. Based on these four variables, we constructed a clinical nomogram in the training cohort, which achieved a C-index of 0.71. In the internal validation cohort, the areas under the receiver operating characteristic curve (AUC-ROC) for predicting 1-, 2-, and 3-year OS were 0.577, 0.721, and 0.730, respectively; in the external validation cohort, the corresponding AUC-ROCs were 0.841, 0.757, and 0.598. Subsequently, each patient’s Rad-score was integrated with these clinical predictors to develop a clinical–radiomics nomogram, which demonstrated a C-index of 0.892. The AUC-ROCs for predicting 1-, 2-, and 3-year OS were 0.791, 0.846, and 0.840 in the internal validation cohort, and 0.863, 0.830, and 0.734 in the external validation cohort.

**Conclusion:**

The clinical–radiomics nomogram demonstrated superior predictive performance for OS compared to the clinical nomogram in inoperable PC patients undergoing CCRT.

## Introduction

Pancreatic cancer (PC) is one of the most prevalent malignancies and constitutes a leading cause of cancer-related mortality, thereby representing a significant threat to human health (1). When permitted, surgical resection is the primary treatment modality for primary pancreatic tumors. However, the majority of PC patients present with advanced-stage disease at diagnosis, and only a minority are eligible for curative resection (2). This leads to an extremely poor prognosis for patients with PC, with a 5-year survival rate of < 5% following diagnosis (3). Continued treatment of PC is important at this time, because the progression of primary tumors leads to morbidity and mortality through invasion of nearby organs and blood vessels. This process is identified as the leading cause of mortality in at least 30% of patients with PC (4, 5). Concurrent chemoradiotherapy (CCRT) provides an option for local disease control (6).

Over the past few decades, there has been a gradual improvement in the clinical outcomes of patients with PC have gradually, largely attributable to the widespread adoption and standardization of radiotherapy, as well as advances in multimodal treatment strategies (7). Prior research have indicated that patients with locally advanced PC who undergo CCRT achieve a significantly higher 1-year survival rate than those treated with chemotherapy alone (8). Additionally, the overall survival (OS) of patients treated with radiotherapy (RT) in combination with gemcitabine increased from 9.2 to 11.1 months compared with that of patients receiving gemcitabine alone (9). Furthermore, dose-response analysis revealed that patients receiving an RT prescription dose of 61 Gy achieved significantly better outcomes than those receiving <61 Gy, with 1-year OS rates of 74.7% versus 60.6%, and 1-year progression-free survival rates of 46.2% versus 30.9%, respectively (10). CCRT improves the prognosis of patients with advanced PC. However, several problems associated with CCRT still remain, such as a high risk of recurrence rate, high mortality rate, and unsatisfactory accuracy of prognosis prediction. These limitations are further compounded by the inability to rapidly and dynamically monitor the tumor properties and changes, as well as unsatisfactory treatment outcomes (11). Therefore, improved efficacy prediction models are required. These current situations show that, in the era of personalized and precision medicine, using more powerful auxiliary models to further optimize the clinical workflow of PC is greatly significance.

A recent proposal has introduced a deep learning methods for synthetic medical image generation, with the objective of enhancing the efficiency of convolutional neural networks in cancer image classification (12). Traditional diagnostic functions of medical imaging can be taken one step forward with the introduction of radiology (13). Radiomics converts medical images into high-throughput, mineable data and automatically extracts quantitative features to augment the estimation of clinical indicators in various malignancies (14). Radiomic analysis is emerging as a promising strategy for predicting cancer risk and cancer recurrence (15, 16). This technology enables the revelation of unique insights into tumor behavior through the integration of multimodal data with clinical, pathological, and genomic information, facilitating the decoding of diverse tissue biology (17, 18). However, limited research has been conducted on risk prediction after chemoradiotherapy for advanced inoperable PC.

Accordingly, our study aimed to apply machine learning strategies to identify a new prognostic model and explore its potential for predicting the efficacy of chemoradiotherapy in patients with unresectable PC, with the goal of minimizing diagnostic errors and improving patient treatment. Personalized precision has considerable potential for improving medical procedures.

## Methods

### Patient selection

**Figure 1:**
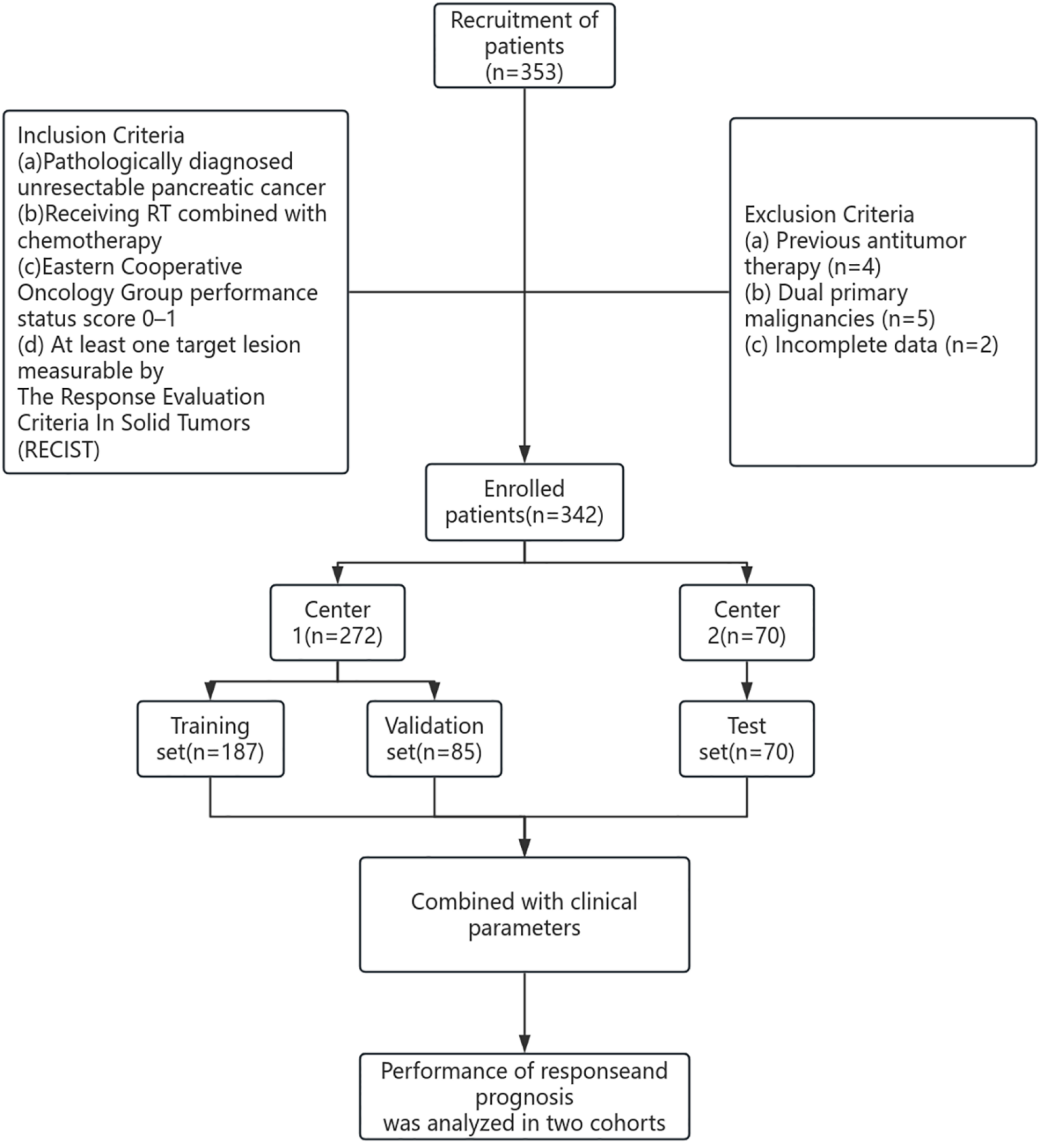
Patient selection flowchart.

The study cohort included patients from two hospitals between 2018 and 2024. The first dataset was obtained from Shandong Province Cancer Hospital (Hospital 1) and divided into training (n=187, 70%) and internal verification (n=85, 30%) datasets. The second dataset was obtained from Chongqing City People’s Hospital (Hospital 2) and served as an external verification set (n=70) (**Figure 1**). The study was approved by the Ethical Review Committees of Shandong Province Cancer Hospital (SDTHEC2024007030) and Chongqing City People’s Hospital (KY S2024-030-01). All patients gave informed consent.

The inclusion criteria were as follows: a) pathologically diagnosed unresectable PC; b) receiving RT combined with chemotherapy; c) Eastern Cooperative Oncology Group performance status score 0–1; d) at least one target lesion measurable by The Response Evaluation Criteria In Solid Tumors (RECIST). Patients exhibiting the following characteristics were excluded: a) previous antitumor therapy; b) dual primary malignancies; or c) incomplete data.

### Treatment and follow-up

All patients received concurrent chemoradiotherapy. Target volumes and critical organs were delineated by at least two senior physicians and medical physicists on contrast-enhanced CT simulation images, with reference to other imaging data such as contrast-enhanced pancreatic MRI. CT scans were performed in the venous phase (60–70 seconds after contrast injection), and the slice thickness was ≤3 mm. Radiotherapy was delivered using volumetric modulated arc therapy (VMAT) and intensity modulated radiation therapy (IMRT). The gross tumor volume (GTV) included radiologically visible pancreatic tumors and metastatic lymph nodes on CT/MRI. The clinical target volume (CTV) encompassed the primary lesion, lymph nodes and perineural invasion sites within approximately 5–10 mm around the pancreatic vasculature, along with lymphatic drainage areas. The planning target volume (PTV) was defined as a 5–10 mm expansion of the CTV. The dose constraints for organs at risk (OARs) followed the RTOG guidelines: Duodenum Dmax ≤55 Gy, V50 ≤10 cm³; Stomach: Dmax ≤55 Gy, V45 ≤75 cm³; Small bowel: Dmax ≤55 Gy, V50 ≤10 cm³; Liver: Dmean ≤25 Gy; Kidneys: Dmean ≤18 Gy, V20 ≤32%; Spinal cord: D1 (dose to 1% volume) ≤45 Gy.

Subsequent patients were generally subjected to regular follow-ups every 3 months during the first year to undergo computed tomography/magnetic resonance imaging (CT/MRI) scans to monitor the effects of RT. Follow-ups were conducted every 6 months in the second year and then annually. Tumor remission was measured using imaging studies in accordance with the RECIST V.1.1 The primary clinical endpoint was OS, defined as the time from the date of initial radiation therapy until mortality or last follow-up.

### Radiomics feature extraction and model construction

**Figure 2:**
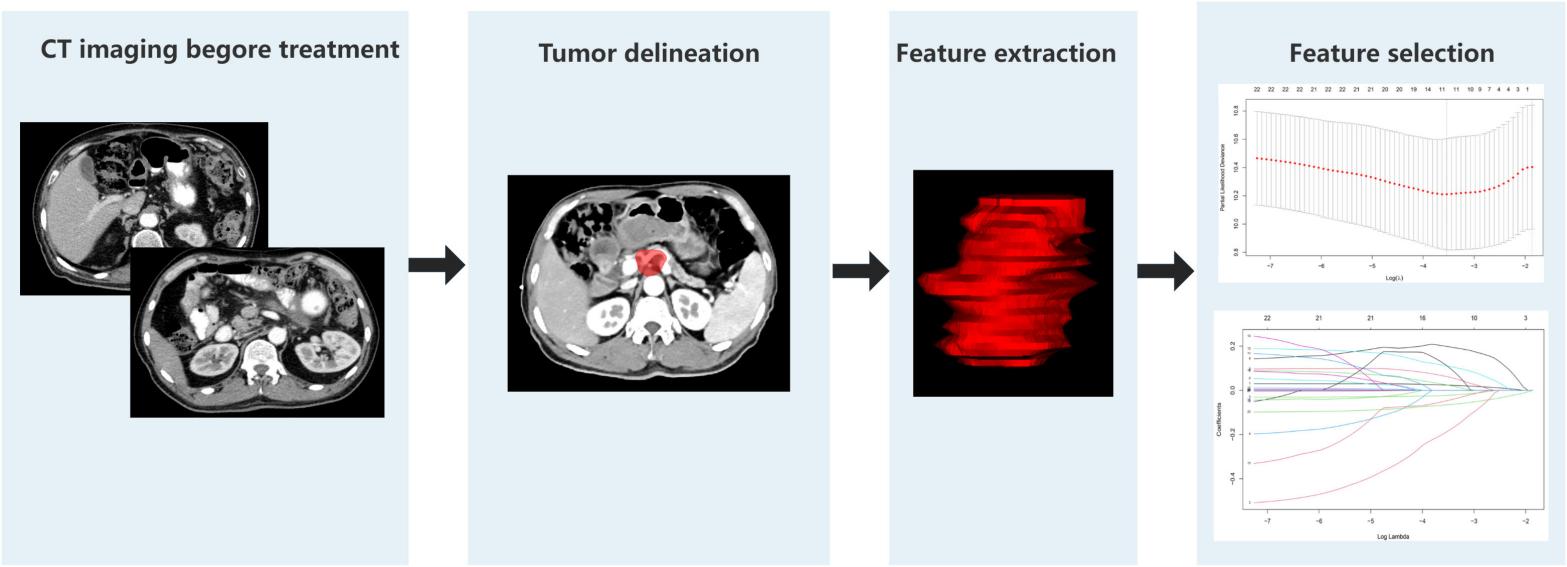
Radiomics pipeline for predicting OS in patients with pancreatic cancer.

A comprehensive set of 1130 radiomic features were extracted from the gross tumor volume in the contrast-enhanced CT scan of each patient with PC using 3D-slicer software. The regions of interest (ROIs) were selected from the primary tumor sites, and the ROI was drawn strictly within the tumor boundaries (**Figure 2**). First, a univariate Cox regression model was used in the training set to select the optimized feature imaging parameters. A total of 272 patients were randomly divided into a training cohort (n = 187) and an internal validation cohort (n = 85) at a 7:3 ratio. In the training cohort, univariate Cox regression was applied to identify optimized imaging features associated with survival. These selected features were subsequently incorporated into the development of 101 different machine-learning radiomics models. These models were constructed by combining 12 different algorithms: StepCox[forward], Ridge, Enet, Random Survival Forest (RSF), StepCox[both], StepCox[backward], CoxBoost, LASSO, Gradient Boosting Machine, plsRcox, SuperPC, and Support Vector Machine. The concordance index (C-index) of each model was calculated in the training, internal validation, and external validation cohorts (n = 70, Chongqing General Hospital). The model with the highest C-index in the training cohort was chosen as the final model, and the corresponding radiomics score (Rad-score) was calculated for each patient based on this model. The codes used in this study are shown in **Supplementary Data Sheet 1**.

### Nomogram construction

In the training cohort, Least Absolute Shrinkage and Selection Operator (LASSO) regression was applied to screen prognostic factors associated with OS. The factors selected by LASSO were then entered into univariate and multivariate Cox proportional-hazards models to identify independent predictors of OS, which were used to construct Nomogram 1 (clinical nomogram). Next, each patient’s Rad-score was combined with the independent clinical predictors to build Nomogram 2 (clinical–radiomics nomogram). Finally, in both the internal and external validation cohorts, we evaluated the predictive performance of these two models by plotting receiver operating characteristic (ROC) curves, calibration curves, and decision curve analysis (DCA) curves. The online dynamic nomogram uses DynNom (v5.1), shiny (v. 1.11.1), rms (v. 8.0 - 0), survivminer (v. 0.5.0), survival (v3.8 - 3), and an rsconnect (v1.5.1). The website platform is hosted using https://docs.posit.co/shinyapps.io/.

### Statistical analysis

For categorical variables, the chi-square test was used for assessment. For continuous baseline variables, the Shapiro–Wilk test was first applied to examine the normality of data distribution. compared using one-way analysis of variance (ANOVA), whereas non-normally using the Kruskal–Wallis test. Additionally, the Kaplan-Meier method was employed to estimate the survival rate, with the log-rank test subsequently utilized to compare survival curves. All analyses were conducted using R software version 3.3.2. A two-sided P value of less than 0.05 was considered to indicate statistically significant.

## Result

### Patient characteristics

This study included a total of 342 patients with PC. The analysis of baseline characteristics is shown in **Table 1**. No significant baseline differences were observed among the training set (n = 187), internal validation group (n = 85), and external validation group (n = 70).

**Table 1:** Patient baseline data analysis table.

Variables	Training cohort (n=187)	Internal validation (n=85)	External validation (n=70)	P	W-value	Shapiro-wilk p-value	Normality
Age (mean ± SD)	61.4 ± 10.5	62.3 ± 11.6	62.1 ± 12.1	0.791	0.993	0.127	Normal
Sex				0.9			
Female	80 (42.8%)	38 (44.7%)	32 (45.7%)				
Male	107 (57.2%)	47 (55.3%)	38 (54.3%)				
BMI (mean ± SD)	22.0 ± 3.7	22.2 ± 3.7	21.9 ± 3.7	0.807	0.996	0.434	Normal
Diabetesmellitus				0.161			
No	147 (78.6%)	75 (88.2%)	56 (80.0%)				
Yes	40 (21.4%)	10 (11.8%)	14 (20.0%)				
Hypertension				0.999			
No	136 (72.7%)	62 (72.9%)	51 (72.9%)				
Yes	51 (27.3%)	23 (27.1%)	19 (27.1%)				
Jaundice				0.394			
No	145 (77.5%)	72 (84.7%)	56 (80.0%)				
Yes	42 (22.5%)	13 (15.3%)	14 (20.0%)				
Abdominal pain				0.313			
No	113 (60.4%)	44 (51.8%)	37 (52.9%)				
Yes	74 (39.6%)	41 (48.2%)	33 (47.1%)				
Stage				0.936			
I	18 (9.63%)	6 (7.06%)	6 (8.57%)				
II	21 (11.2%)	7 (8.24%)	6 (8.57%)				
III	47 (25.1%)	26 (30.6%)	19 (27.1%)				
IV	101 (54.0%)	46 (54.1%)	39 (55.7%)				
T				0.935			
1	8 (4.28%)	2 (2.35%)	2 (2.86%)				
2	40 (21.4%)	18 (21.2%)	13 (18.6%)				
3	33 (17.6%)	11 (12.9%)	10 (14.3%)				
4	82 (43.9%)	43 (50.6%)	35 (50.0%)				
x	24 (12.8%)	11 (12.9%)	10 (14.3%)				
N				0.983			
0	86 (46.0%)	36 (42.4%)	31 (44.3%)				
1	61 (32.6%)	31 (36.5%)	24 (34.3%)				
2	14 (7.49%)	7 (8.24%)	7 (10.0%)				
x	26 (13.9%)	11 (12.9%)	8 (11.4%)				
M				0.969			
0	86 (46.0%)	39 (45.9%)	31 (44.3%)				
1	101 (54.0%)	46 (54.1%)	39 (55.7%)				
Liverm				0.516			
No	129 (69.0%)	53 (62.4%)	45 (64.3%)				
Yes	58 (31.0%)	32 (37.6%)	25 (35.7%)				
Lungm				0.092			
No	176 (94.1%)	77 (90.6%)	60 (85.7%)				
Yes	11 (5.88%)	8 (9.41%)	10 (14.3%)				
Otherm				0.933			
No	132 (70.6%)	60 (70.6%)	51 (72.9%)				
Yes	55 (29.4%)	25 (29.4%)	19 (27.1%)				
Size (M, IQR)	3.8 (3.0 - 5.0)	4.2 (3.3 - 5.1)	3.5 (2.9 - 5.1)	0.396	0.927	< 0.001	Non-normal
White blood cell (M, IQR)	5.6 (4.4 - 7.1)	5.7 (4.4 - 7.0)	5.7 (4.6 - 7.2)	0.963	0.693	< 0.001	Non-normal
Lymphocyte (M, IQR)	1.4 (1.1 - 1.7)	1.3 (1.0 - 1.6)	1.3 (1.0 - 1.6)	0.368	0.801	< 0.001	Non-normal
Neutrophil (M, IQR)	3.4 (2.4 - 4.9)	3.8 (2.6 - 5.0)	3.6 (2.7 - 5.1)	0.949	0.604	< 0.001	Non-normal
Neutrophil–lymphocyte ratio (M, IQR)	2.5 (1.6 - 3.8)	3.0 (1.9 - 4.3)	3.0 (1.8 - 4.5)	0.92	0.642	< 0.001	Non-normal
Hemoglobin (mean ± SD)	122.0 ± 17.4	121.0 ± 17.4	122.0 ± 18.1	0.741	0.997	0.6741	Normal
Blood platelet (M, IQR)	217.0 (160.5 - 271.5)	236.0 (171.0 - 302.0)	219.5 (160.8 - 284.8)	0.733	0.924	< 0.001	Non-normal
Total bilirubin (M, IQR)	11.9 (8.0 - 23.2)	10.7 (7.2 - 20.2)	9.3 (6.7 - 13.7)	0.516	0.472	< 0.001	Non-normal
Alanine aminotransferase (M, IQR)	25.1 (15.4 - 68.3)	22.6 (14.3 - 53.3)	20.3 (12.2 - 47.8)	0.142	0.527	< 0.001	Non-normal
Aspartate transaminase (M, IQR)	25.3 (17.9 - 46.2)	24.3 (17.1 - 44.5)	23.1 (16.1 - 37.8)	0.477	0.596	< 0.001	Non-normal
Albumin (mean ± SD)	41.9 ± 5.3	41.3 ± 4.8	42.1 ± 6.5	0.623	0.935	< 0.001	Non-normal
CA199 (M, IQR)	167.6 (38.6 - 701.5)	294 (53.3 - 1212.0)	276 (39.6 - 1313.3)	0.441	0.531	< 0.001	Non-normal

### OS

In the training set, internal validation group, and external validation groups, the median OS was 20.3, 22.2, and 21.5 months respectively, while the 3-year survival rates were 36.9%, 30.4%, and 28.7%, respectively (P = 0.9, **Supplementary Figure 1**).

### Radiomics models

Nine risk indicators were screened from 1,130 data using single-factor Cox. The selected characteristics were as follows:

wavelet.LHL.firstorder.Range
wavelet.LHL.glszm.SizeZoneNonUniformityNormalized
wavelet.HLH.glszm.GrayLevelNonUniformity
wavelet.HHH.gldm.LowGrayLevelEmphasis
wavelet.HHH.glrlm.LowGrayLevelRunEmphasis
wavelet.HHH.glrlm.ShortRunLowGrayLevelEmphasis
wavelet.HHH.glszm.GrayLevelNonUniformity
wavelet.HHH.glszm.SmallAreaEmphasis
wavelet.HHH.glszm.ZoneEntropy

**Figure 3:**
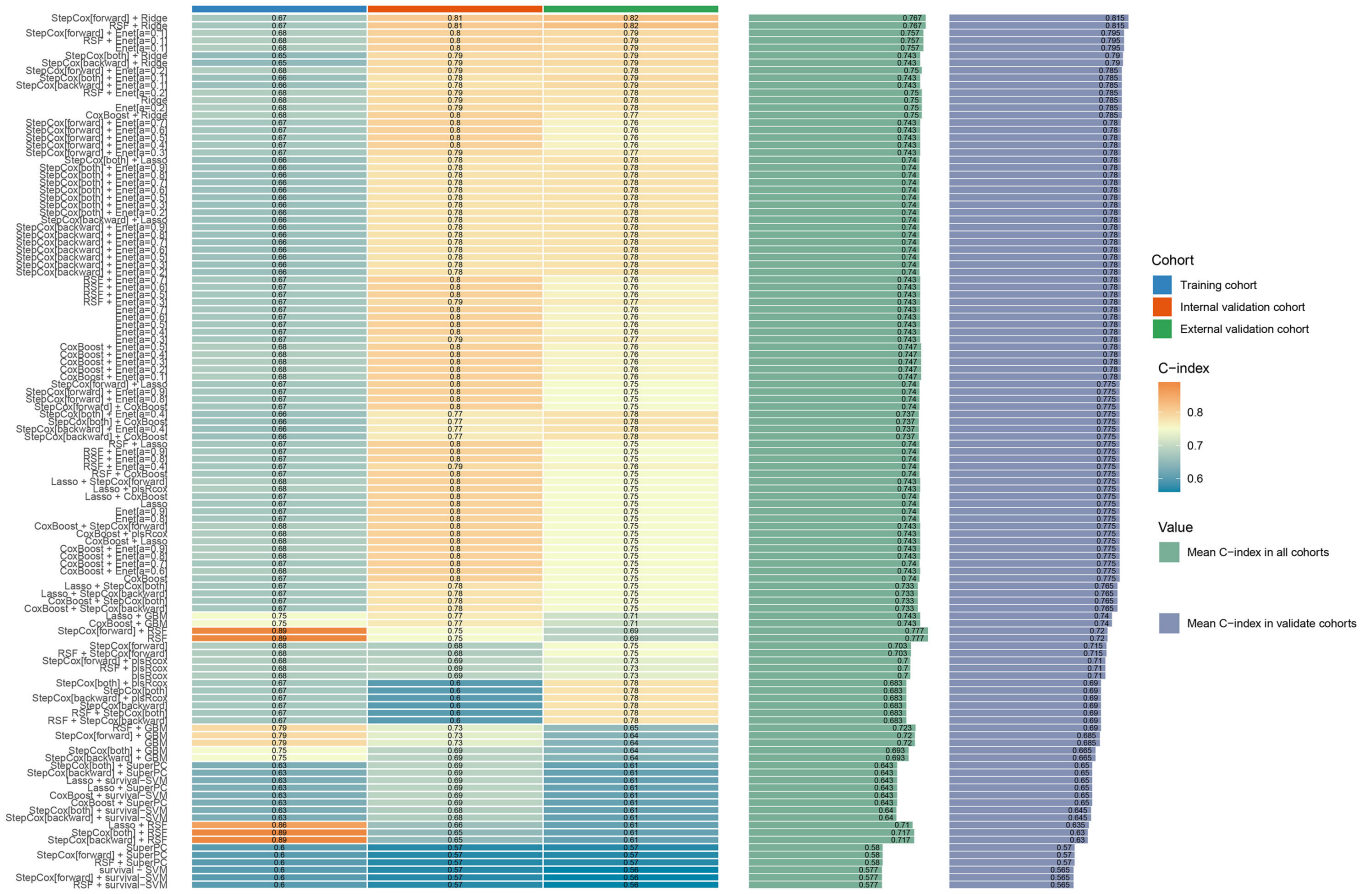
Machine learning model evaluation and correlation curve analysis: The consistency index (C index) for different machine learning models.

Based on these nine risk indicators, 101 machine learning models were constructed by combining 12 models: StepCox[forward], Ridge, Enet, random survival forest (RSF), StepCox[both], StepCox[backward], CoxBoost, LASSO, gradient boosting machine, plsRcox, SuperPC, and survival support vector machine (**Figure 3**). The results revealed that StepCox[forward]+ RSF had the highest C-index of 0.89 (**Supplementary Figure 2**). The Rad-score for each patient was determined by means of calculation using the model. As shown in **Supplementary Figure 3**, three sets of survival curves are presented based on the cutoff value of the median risk score. These results indicate that the image model demonstrates a satisfactory predictive capacity.

### Clinical prognostic factor selection: LASSO + Cox

In the training set, LASSO regression selected the following 11 variables associated with OS: Age, body mass index (BMI), sex, T stage, N stage, lymphocyte count, neutrophil count, CA19–9 level, clinical stage, tumor size, and serum albumin (**Supplementary Figures 4A, B**). Subsequently, the 11 prognostic factors were entered into univariate and multivariate Cox regression analyses, which confirmed that age, clinical stage, tumor size, and albumin level were independent prognostic factors (**Table 2**).

**Table 2:** Univariate and multivariate Cox regression models for overall survival.

Characteristic	Univariate analysis			Multivariate analysis		
	HR	95% CI	P value	HR	95% CI	P value
Age	1.03	1.01-1.06	**0.004**	1.03	1.01-1.06	**0.014**
BMI	0.94	0.88-1.00	**0.045**	0.97	0.91-1.04	0.398
Sex						
Female	1.000					
Male	0.60	0.38-0.94	**0.027**	0.69	0.43-1.10	0.117
T						
1	1.000					
2	3.02	0.69-13.16	0.142	4.09	0.88-19.03	0.073
3	2.75	0.62-12.21	0.184	1.65	0.36-7.57	0.521
4	3.42	0.82-14.29	0.091	1.50	0.32-7.06	0.607
x	5.18	1.12-23.85	**0.035**	3.82	0.74-19.78	0.111
N						
0	1.000					
1	1.16	0.70-1.92	0.558			
2	0.98	0.35-2.77	0.975			
x	1.95	0.98-3.88	0.056			
Lymphocyte	1.12	0.84-1.50	0.426			
Neutrophil	0.97	0.89-1.05	0.420			
CA199	1.00	1.00-1.00	0.071			
Stage						
1	1.000					
2	1.05	0.37-3.01	0.925	2.00	0.59-6.76	0.266
3	1.93	0.82-4.52	0.132	4.18	1.26-13.88	**0.019**
4	2.34	1.04-5.23	**0.039**	4.04	1.44-11.37	**0.008**
Size	1.12	1.01-1.24	**0.029**	1.16	1.02-1.32	**0.020**
Albumin	0.92	0.88-0.96	**0.001**	0.91	0.87-0.96	**0.001**

The bold values indicate statistical significance with P < 0.05.

### Clinical nomogram and clinical–radiomics nomogram

**Figure 4:**
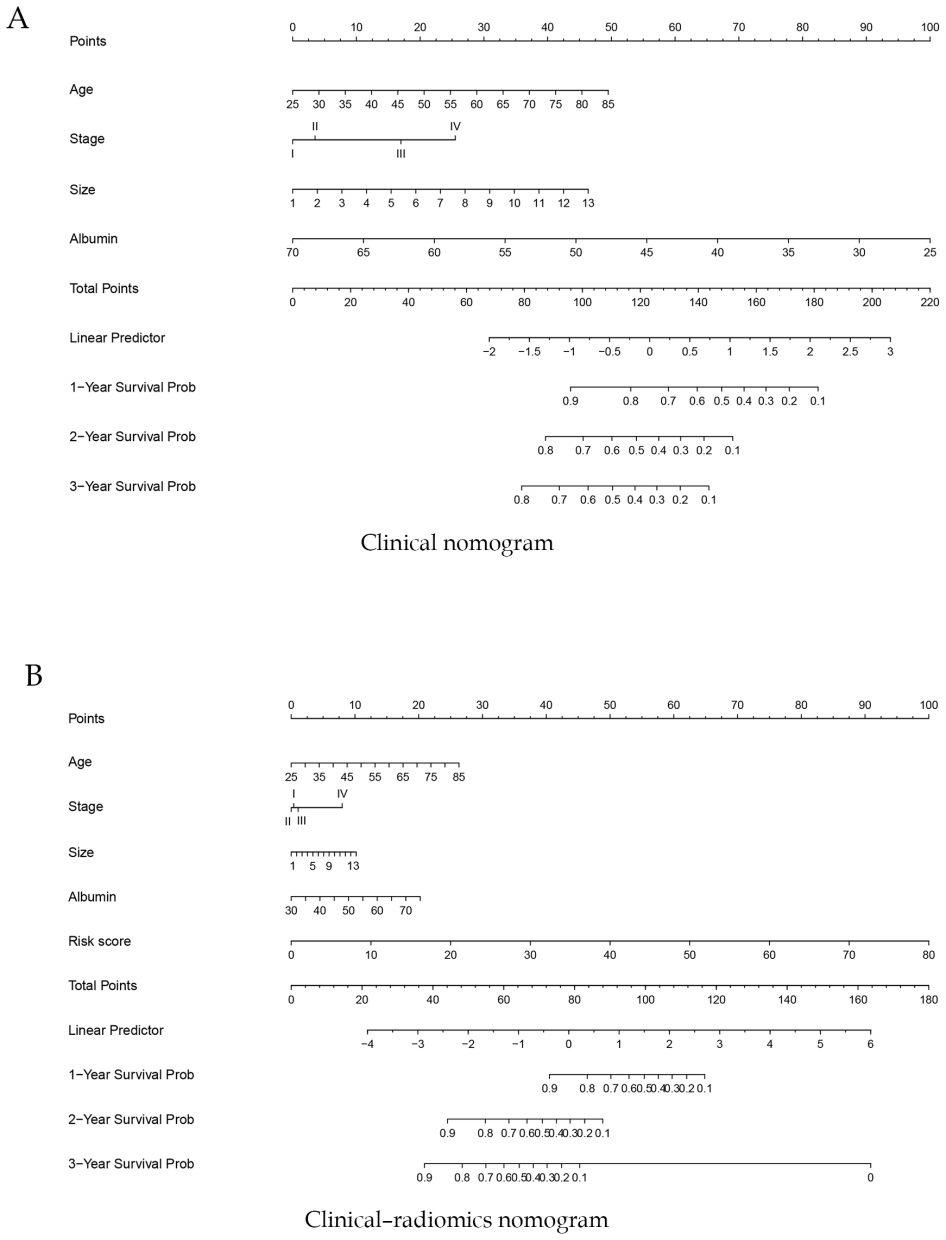
Construction of a clinical nomogram **(A)** and a clinical–radiomics nomogram **(B)** for predicting the prognosis of pancreatic cancer based on clinical characteristics in the training cohort.

In the training cohort, a clinical nomogram was first developed based on independent clinical prognostic factors identified by multivariate Cox analysis (**Figure 4A**). Subsequently, each patient’s radiomics signature (Rad-score) was integrated with these independent clinical predictors to construct a clinical–radiomics nomogram (**Figure 4B**). At the same time, we have developed online dynamic nomograms for real-time use by clinicians. One of them is an online nomogram for clinical model (https://yanzeli95.shinyapps.io/DynNomapp_no_risk_score/). The other is an online nomogram for clinical-radiomics model (https://yanzeli95.shinyapps.io/DynNomapp_risk_score/).

**Figure 5:**
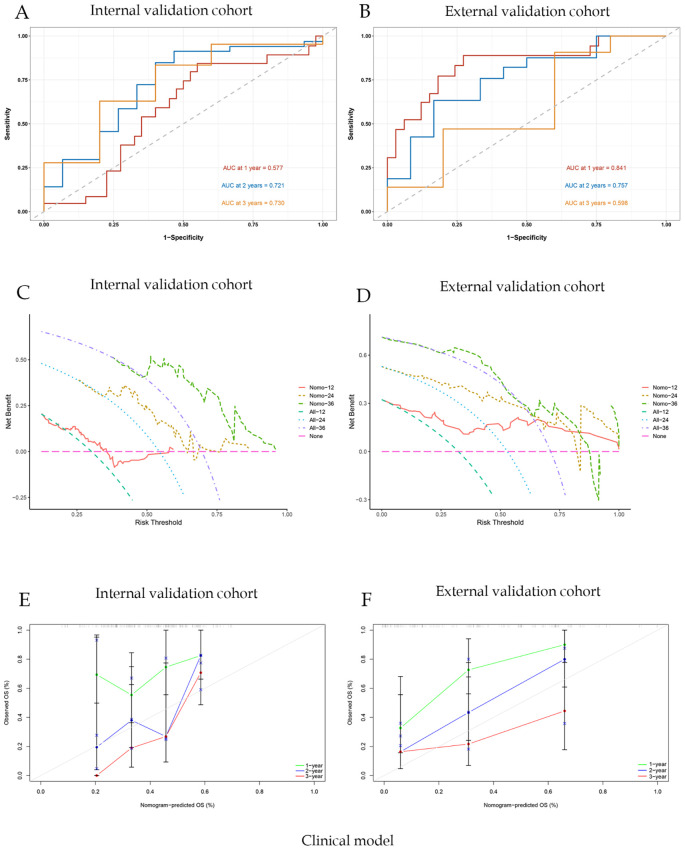
Clinical model evaluation and analysis of correlation curves; **(A)** ROC curve of nomogram model with AUC of internal validation cohort; **(B)** ROC curve of nomogram model with AUC of external validation cohort; **(C)**Nomogram model clinical decision curve (DCA) of internal validation cohort; **(D)** Nomogram model clinical decision curve (DCA) of external validation cohort; **(E)** Calibration curves of internal validation cohort; **(F)** Calibration curves of external validation cohort.

For the clinical nomogram, the area under the ROC curves (AUC-ROC) for predicting 1-, 2-, and 3-year OS were 0.577, 0.721, and 0.730 in the internal validation cohort (**Figure 5A**), and 0.841, 0.757, and 0.598 in the external validation cohort (**Figure 5B**). DCA showed that the clinical nomogram provided greater net benefit than treat-all or treat-none strategies across a range of threshold probabilities in both validation cohorts (**Figures 5C, D**). Calibration plots demonstrated close agreement between predicted and observed survival, confirming the model’s stability (**Figures 5E, F**). The C-index of the nomogram was 0.71.

**Figure 6:**
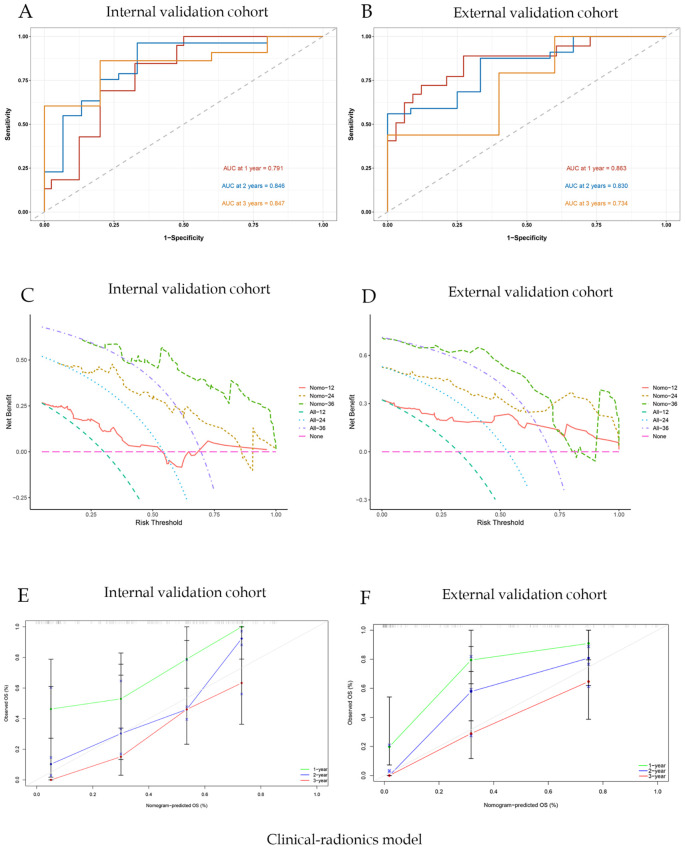
Joint clinic-radionics model evaluation and correlation curve analysis; **(A)** ROC curve of nomogram model with AUC of internal validation cohort; **(B)** ROC curve of nomogram model with AUC of external validation cohort; **(C)** Nomogram model clinical decision curve (DCA) of internal validation cohort; **(D)** Nomogram model clinical decision curve (DCA) of external validation cohort; **(E)** Calibration curves of internal validation cohort; **(F)** Calibration curves of external validation cohort.

For the clinical-radiomics nomogram, the AUC-ROC for predicting 1-, 2-, and 3-year OS were 0.791, 0.846, and 0.840 in the internal validation cohort (**Figure 6A**), and 0.863, 0.830, and 0.734 in the external validation cohort (**Figure 6B**). DCA showed net benefit over treat-all/none strategies across thresholds (**Figures 6B, E**), and calibration plots confirmed close agreement between predicted and observed survival (**Figures 6C, F**). The C-index of the nomogram was 0.892. And the briefer scores of the two models are shown in **Table 3**.

**Table 3:** The brifer scores of the two models.

		1-year	2-year	3-year
Clinical model	Internal verification set	0.208	0.183	0.18
	external verification set	0.142	0.173	0.225
Radiology combined clinical model	Internal verification set	0.158	0.14	0.139
	external verification set	0.125	0.147	0.179

## Discussion

To our knowledge, this is the first clinical-radiomic prognostic risk model of survival to chemoradiotherapy in patients with inoperable PC, which may be a new prognostic imaging biomarker for PC. PC has the highest mortality rate among all malignant tumors worldwide (19). Despite significant advances in medical technology, the prognosis of PC has improved significantly (20). However, the overall prognosis for patients diagnosed with PC continues to be unfavorable, representing a significant threat to their survival (21, 22). We combined clinical and radiomics features, optimizing the screening and combination of radiomics parameters related to the prognosis of PC using a variety of machine learning methods, which improved the accuracy of the prediction results. Additionally, a significant machine learning radiomics prognostic model was developed to predict the OS to chemoradiotherapy in patients diagnosed with inoperable PC.

Chemoradiotherapy improves the prognosis of patients diagnosed with inoperable PC (23). Therefore, predicting the efficacy of chemoradiotherapy has become a top priority. Previous studies have demonstrated that factors such as patients’ age, tumor differentiation, tumor size, serum alkaline phosphatase, albumin level, and CA 19–9 can serve as independent predictors of PC prognosis (24). Several predictive models have been developed for PC. A retrospective study leveraging both the Surveillance, Epidemiology, and End Results(SEER) database and a Chinese cohort developed a prognostic nomogram for PC based on clinical characteristics. This nomogram demonstrated AUC for 1-, 3-, and 5-year survival rates of 0.71, 0.82, and 0.81, respectively (25). Based on an analysis of clinical parameters and DNA methylation risk scores, Deng et al. established and validated a nomogram with 1-, 2-, and 3-year AUCs of 0.899, 0.765, and 0.766, respectively (26). Our findings that the 1-, 2-, and 3-year AUCs of the clinical model constructed to predict inoperable PC survival were 0.577, 0.721, and 0.730, respectively. Although the predictive power of clinical models is acceptable, these indicators cannot identify patients with a high probability of recurrence or a poor prognosis after treatment through subtle heterogeneous changes within tumors (27, 28).

However, few studies have used machine learning for prognostic analysis of PC. Some researchers have constructed a prognostic model to evaluate the prognosis the outcomes of patients with PC liver metastases who are undergoing chemoimmunotherapy based on magnetic resonance features and clinical data. The nomogram generated from this model achieved an AUC of 0.770 for predicting 1-year OS (29). Although this analysis demonstrated the usefulness of clinical and other models in the diagnosis of PC, it did not explore the ability of radiomics to predict prognosis. A recent retrospective case-control research using radiomics data from CT scans to evaluate PC health indicated the merits of radiomics. However, this study did not combine survival benefit outcomes (30).

In this study, we tested a wide range of machine learning algorithms to construct radiomics-based prognostic models for hepatocellular carcinoma. The rationale for employing multiple algorithms lies in the distinct strengths and limitations of each approach (31). For example, Cox-based models offer strong interpretability and clinical relevance but may oversimplify complex nonlinear relationships. Tree-based ensemble methods, such as random survival forests, are capable of capturing high-order interactions and nonlinear effects, yet may lack transparency. Gradient boosting algorithms, such as XGBoost and LightGBM, often demonstrate superior predictive accuracy and robustness in high-dimensional data but require careful tuning to avoid overfitting. Similarly, support vector machines (SVM) and neural network–based models can handle complex feature spaces but may be less intuitive for clinical translation. By systematically comparing these algorithms across training, internal, and external validation cohorts using the concordance index, we ensured that the final selected model achieved both optimal performance and generalizability. This comparative approach not only highlights the variability in algorithmic performance but also strengthens confidence in the robustness of the chosen model. Ultimately, the use of diverse machine learning strategies allowed us to balance predictive accuracy with interpretability, ensuring the proposed radiomics score is both scientifically sound and clinically applicable. And we employed both stepwise Cox and RSF to build and evaluate prognostic models for PC. Stepwise Cox was chosen for its strong clinical interpretability and ability to identify key prognostic variables while maintaining a parsimonious model structure. In contrast, RSF offers distinct advantages in capturing complex, nonlinear relationships and interactions among variables without relying on the proportional hazards assumption (32). By combining these two approaches, we were able to leverage the complementary strengths of interpretability and predictive accuracy. This dual strategy not only provides robust survival prediction but also ensures that the results remain clinically meaningful and applicable to individualized treatment planning.

Patients with carcinoma of pancreatic head undergoing radical resection were analyzed with respect to the ability of radiomics and clinical data to predict OS, demonstrating that CT scans revealed shorter disease-free survival for portal vein-stage hypodense PC (33). Study has used purely clinical variables to establish clinical nomograms for pancreatic cancer or other types of cancer. For example, there are studies that focus on clinical data from patients with pancreatic cancer, using clinical variables (such as tumor stage, CA19–9 levels, albumin levels, etc.) to predict patients ‘OS. These studies have generally shown that pure clinical nomograms can provide some predictive power, but their predictive accuracy is often limited by clinical variables (34). Parr et al. performed a retrospective study involving 74 patients with marginally resectable PC who received stereotactic RT. They compared the performances of clinical, radiomic, and combination models in predicting outcomes. The mean AUC of the three models were 0.66, 0.78, and 0.77, respectively. Their findings indicated that the radiomics feature-based model could better predict the OS and recurrence rate of PC than the clinical feature-based model (35). This study aligns with ours in indicating the ability of single and combined models to provide critical information about pancreatic tissues. In this study, the C-index of the clinical model was 0.71, while the C-index of the combined model increased to 0.892, indicating a significant improvement in the accuracy of the combined model in predicting OS. This result suggests that the combined model can better integrate clinical variables and radiologic characteristics, thereby providing more accurate prognostic assessment. Although these predictive models can assess patient survival, their predictive performance remains suboptimal. In our study, the 1-, 2-, and 3-year AUCs of the clinical-radiomics model were 0.791, 0.846, and 0.847, respectively. Our newly constructed joint model had better predictive performance for prognosis than the models in other studies. In addition, using Cox regression models for variable screening (such as LASSO) is also an effective method to suppress overfitting. It reduces the complexity of the model by selecting variables and avoids overfitting problems. Future research can further verify the application effect of this model in different populations and clinical settings by increasing the sample size and conducting more extensive external validation. It can effectively reduce the possibility of over-fitting and improve the stability of the model.

The clinical–radiomics nomogram exhibited a higher C-index and AUC–ROC compared with the clinical nomogram. This indicates that the combined model had stronger predictive stability and validity than the single models, and the clinical radiomics combined model had a better prognosis in this study. This may be due to the ability of imaging features to capture microcarcinomas that are not visible to the naked eye and tumor cells that are missing from the margins of the target. The radiomics model in this study predicted a lower proportion of cancer mortality in low-risk patients than in high-risk patients, which was difficult to predict using clinical laboratory data.

The joint nomogram constructed in this study has the capacity to accurately predict the prognostic status of PC by integrating factors, such as physiological indicators, medical history, biochemical indicators, tumor stage, and imaging characteristics. Clinicians can perform a preliminary assessment of the patient’s prognosis before treatment based on predicted results of the nomogram, provide patients with more accurate disease information and treatment recommendations, and enhance their confidence and compliance with the treatment plan. Thus, a more personalized treatment plan can be developed, which can effectively enhance both the survival rate and the quality of life of patients. Radiomic characteristics have great potential in revealing the tumor microenvironment, cellular heterogeneity, and tumor invasiveness. Combining these biological characteristics with clinical prognostic variables can help us understand tumor behavior more comprehensively and further enhance the clinical application value of prognostic models. In addition, with the continuous development of artificial intelligence and machine learning technology, the application of radiology will play an increasingly important role in cancer diagnosis, staging and treatment decisions.

Radiomics data has the potential to facilitate a more profound comprehension of tumor behavior, thereby enabling the creation of enhanced predictive models (36). In this study, a combined clinical radiomics prediction model was compared with a traditional clinical prediction model in order to evaluate their abilities to predict PC prognosis. This study has several advantages. Due to the large sample size and internal and external validation, our study ensured credibility and authenticity. However, this study has some limitations. Firstly, the use of retrospective data may have introduced data bias. Secondly, because this study included only Chinese patients, the generalizability of the results to other countries is limited. However, despite these issues, the machine learning-based prognostic models were specifically developed to predict the survival prognostic survival in patients with inoperable PC. All parameters necessary for this model were derived from routine clinical practice and are readily applicable by clinicians to inform comprehensive treatment plans for patients with inoperable PC. This enables early intervention to prevent and address potential adverse clinical events.

## Conclusion

The clinical–radiomics nomogram demonstrated superior predictive performance for OS compared to the clinical nomogram in inoperable PC patients undergoing CCRT.

## Data Availability

The raw data supporting the conclusions of this article will be made available by the authors, without undue reservation.
