# Supplementary figures and images for: A nomogram-based radiomics for predicting survival to concurrent chemoradiotherapy in inoperable pancreatic cancer: a dual-center cohort study
Source: Front Immunol. 2025 Oct 23;16:1655803. doi: 10.3389/fimmu.2025.1655803 (PMC12589057; doi:10.3389/fimmu.2025.1655803)

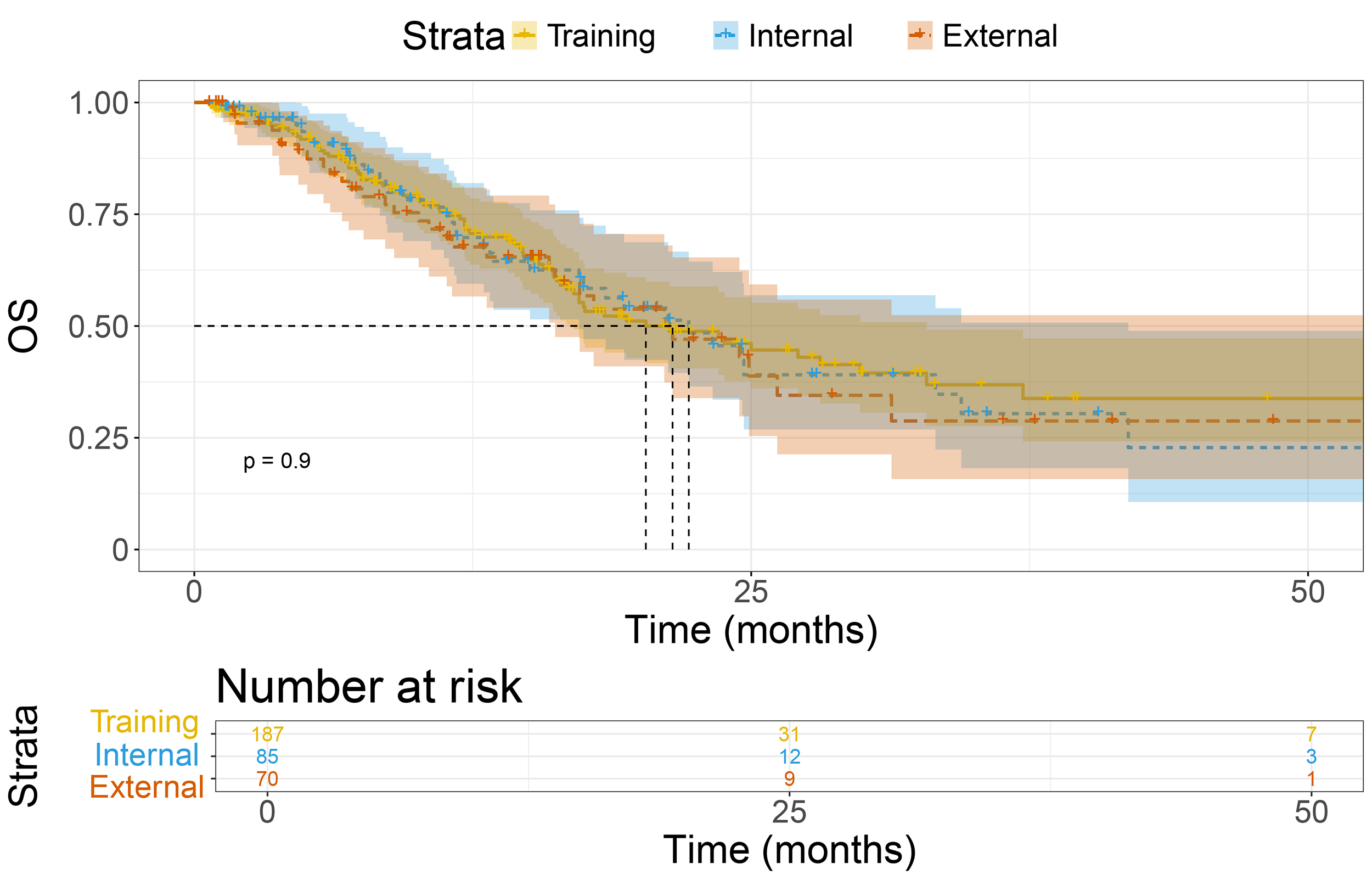

Supplement: Supplementary file 2 [file Image1.tif]

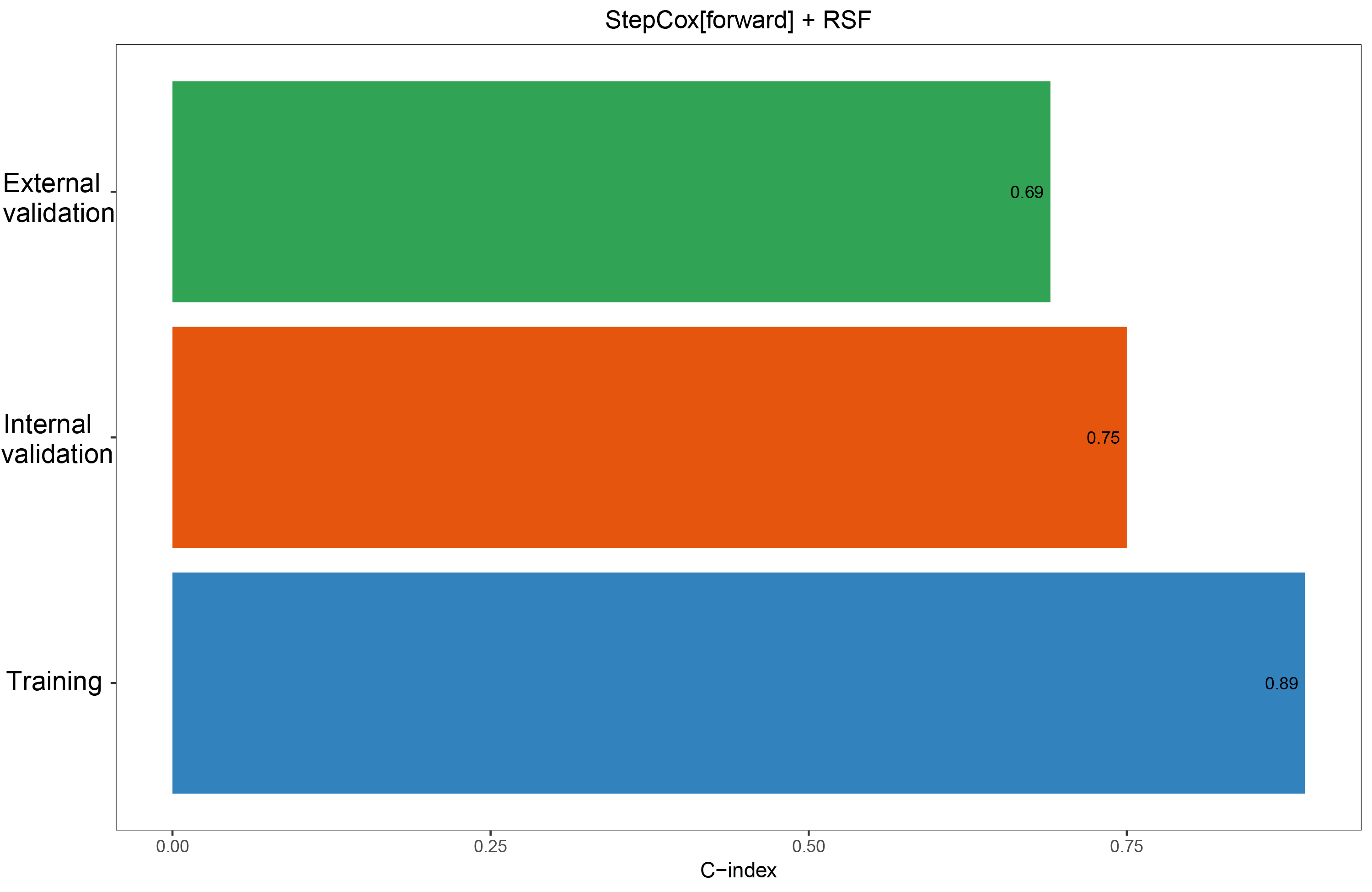

Supplement: Supplementary file 3 [file Image2.tif]

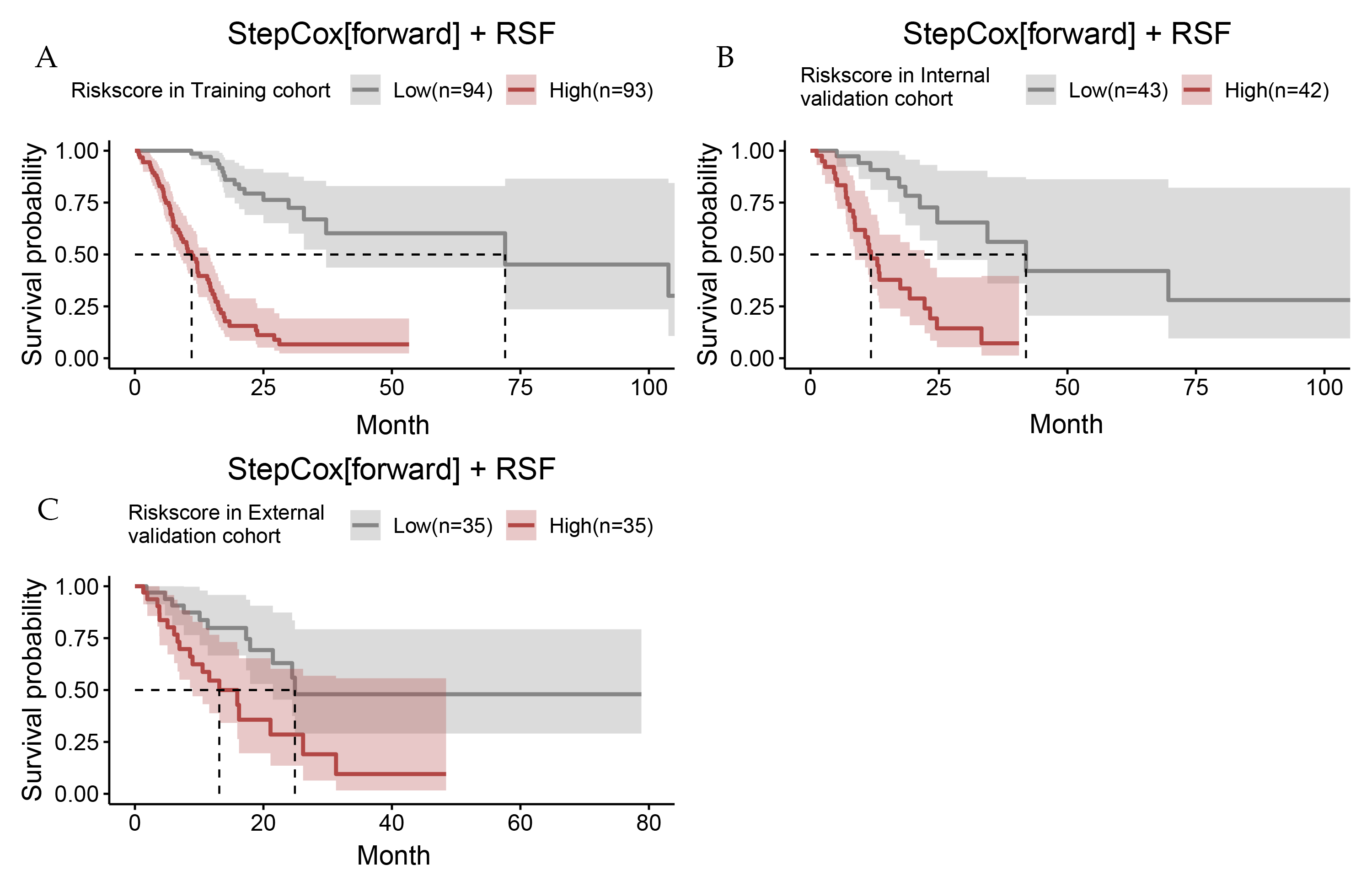

Supplement: Supplementary file 4 [file Image3.tif]

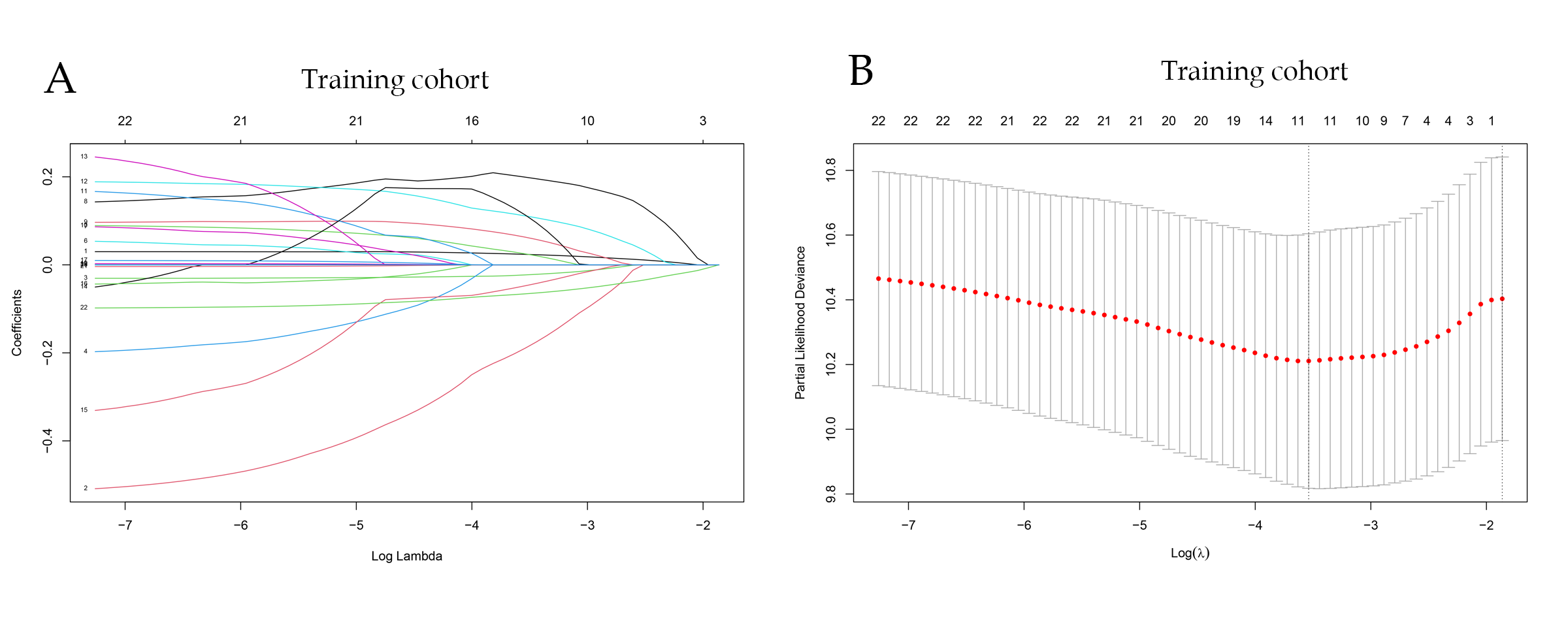

Supplement: Supplementary file 5 [file Image4.tif]
